# Bilateral remote ischemic conditioning in children: A two-center, double-blind, randomized controlled trial in young children undergoing cardiac surgery

**DOI:** 10.1016/j.xjon.2024.02.018

**Published:** 2024-03-05

**Authors:** Nigel E. Drury, Carin van Doorn, Rebecca L. Woolley, Rebecca J. Amos-Hirst, Rehana Bi, Collette M. Spencer, Kevin P. Morris, James Montgomerie, John Stickley, Adrian Crucean, Alicia Gill, Matt Hill, Ralf J.M. Weber, Lukas Najdekr, Andris Jankevics, Andrew D. Southam, Gavin R. Lloyd, Osama Jaber, Imre Kassai, Giuseppe Pelella, Natasha E. Khan, Phil Botha, David J. Barron, Melanie Madhani, Warwick B. Dunn, Natalie J. Ives, Paulus Kirchhof, Timothy J. Jones, Bilateral Remote Ischemic Conditioning in Children Trial Investigators, Edmund D. Carver, Alistair J. Cranston, Fraser Harban, Vasco Laginha Rolo, Ritchie Marcus, Anthony Moriarty, Raju Reddy, Susanna N. Ritchie-McLean, Monica A. Stokes, Ayngara Thillaivasan, Nandlal Bhatia, Carol Bodlani, Wendy Lim, Joe Mellor, Jutta Scheffczik

**Affiliations:** a Institute of Cardiovascular Sciences, University of Birmingham, Birmingham, United Kingdom; b Department of Paediatric Cardiac Surgery, Birmingham Children's Hospital, Birmingham, United Kingdom; c Department of Congenital Cardiac Surgery, Leeds Teaching Hospitals NHS Trust, Leeds, United Kingdom; d Birmingham Clinical Trials Unit, University of Birmingham, Birmingham, United Kingdom; e Institute of Applied Health Research, University of Birmingham, Birmingham, United Kingdom; f Department of Paediatric Intensive Care, Birmingham Children's Hospital, Birmingham, United Kingdom; g Department of Paediatric Cardiac Anesthesia, Birmingham Children's Hospital, Birmingham, United Kingdom; h Phenome Centre Birmingham, School of Biosciences, University of Birmingham, Birmingham, United Kingdom; i Division of Cardiovascular Surgery, Hospital for Sick Children, Toronto, Canada; j Department of Surgery, University of Toronto, Toronto, Canada; k Department of Biochemistry and Systems Biology, Institute of Systems, Molecular, and Integrative Biology, University of Liverpool, Liverpool, United Kingdom; l Department of Cardiology, University Heart and Vascular Centre, UKE Hamburg, Hamburg, Germany; m German Centre for Cardiovascular Research (DZHK), Partner Site Hamburg/Kiel/Lübeck, Hamburg, Germany

**Keywords:** clinical trial, cyanosis, myocardial protection, pediatric cardiac surgery, remote ischemic preconditioning, tetralogy of Fallot

## Abstract

**Objective:**

The study objective was to determine whether adequately delivered bilateral remote ischemic preconditioning is cardioprotective in young children undergoing surgery for 2 common congenital heart defects with or without cyanosis.

**Methods:**

We performed a prospective, double-blind, randomized controlled trial at 2 centers in the United Kingdom. Children aged 3 to 36 months undergoing tetralogy of Fallot repair or ventricular septal defect closure were randomized 1:1 to receive bilateral preconditioning or sham intervention. Participants were followed up until hospital discharge or 30 days. The primary outcome was area under the curve for high-sensitivity troponin-T in the first 24 hours after surgery, analyzed by intention-to-treat. Right atrial biopsies were obtained in selected participants.

**Results:**

Between October 2016 and December 2020, 120 eligible children were randomized to receive bilateral preconditioning (n = 60) or sham intervention (n = 60). The primary outcome, area under the curve for high-sensitivity troponin-T, was higher in the preconditioning group (mean: 70.0 ± 50.9 μg/L/h, n = 56) than in controls (mean: 55.6 ± 30.1 μg/L/h, n = 58) (mean difference, 13.2 μg/L/h; 95% CI, 0.5-25.8; *P* = .04). Subgroup analyses did not show a differential treatment effect by oxygen saturations (p_interaction =_ .25), but there was evidence of a differential effect by underlying defect (p_interaction =_ .04). Secondary outcomes and myocardial metabolism, quantified in atrial biopsies, were not different between randomized groups.

**Conclusions:**

Bilateral remote ischemic preconditioning does not attenuate myocardial injury in children undergoing surgical repair for congenital heart defects, and there was evidence of potential harm in unstented tetralogy of Fallot. The routine use of remote ischemic preconditioning cannot be recommended for myocardial protection during pediatric cardiac surgery.

## Graphical abstract

**Figure:**
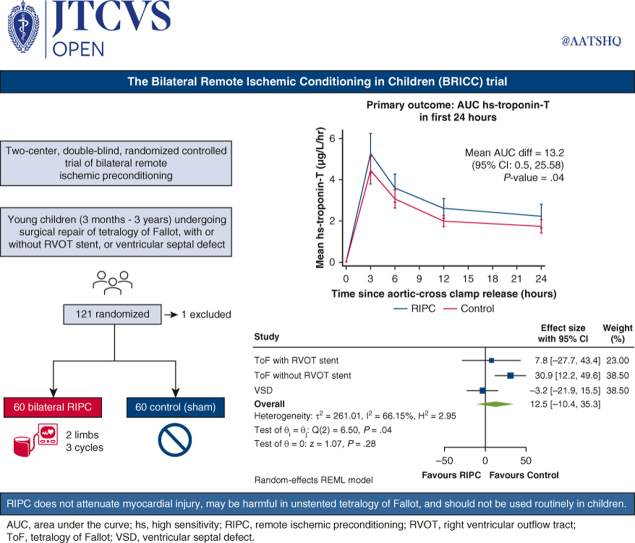

**Figure:**
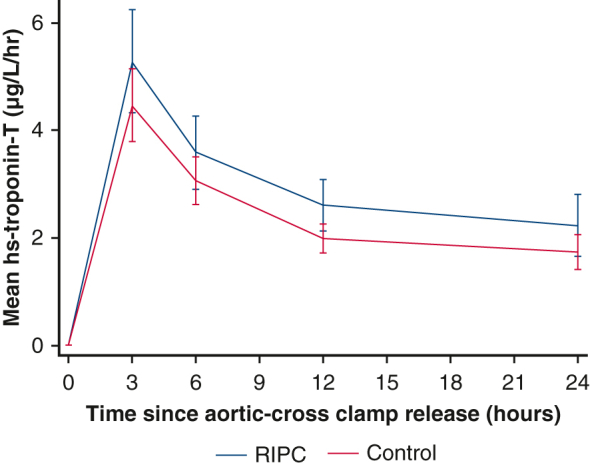
Mean hs-troponin-T release in the first 24 hours after surgery by treatment group.

**Central Message**

RIPC does not improve myocardial protection in young children undergoing surgical repair of common congenital heart defects. It may cause harm and should not be used routinely.

**Perspective**

Previous trials of RIPC in children undergoing cardiac surgery have shown mixed results, and their designs are criticized. We found that adequately delivered, bilateral preconditioning did not attenuate myocardial injury and may be harmful in those with unstented TOF. Its routine use cannot be recommended, and alternative protective strategies should be sought.

Myocardial protection against ischemic-reperfusion injury is a key determinant of heart function and outcome after cardiac surgery in children.^1^ With current strategies, myocardial injury occurs routinely after aortic crossclamping, as quantified by an increase in circulating troponin in the first 24 hours.^2^ This myocardial damage frequently impairs ventricular function, which may manifest as low cardiac output requiring inotropic support. This is a major cause of morbidity and death in the early postoperative period,^1^^,^^3^ and children with preoperative cyanosis may be more vulnerable than acyanotic children.^4^^,^^5^

Remote ischemic preconditioning (RIPC), the application of brief, nonlethal cycles of ischemia and reperfusion to a distant organ or tissue, is a simple, low-risk, and readily available technique that when delivered immediately before surgery may improve myocardial protection. Previous trials of RIPC in children undergoing cardiac surgery have shown mixed results^6^, ^7^, ^8^, ^9^, ^10^, ^11^, ^12^, ^13^, ^14^ and have been criticized for a potentially inadequate stimulus; a manual sphygmomanometer may allow subclinical reperfusion during the ischemic phase,^12^ and propofol anesthesia has been suggested to interfere with the preconditioning pathway.^15^ In addition, they have not evaluated the effects of preoperative cyanosis on RIPC^16^ and have only applied the cuff to a single limb, potentially delivering a subtherapeutic stimulus in young children with a lower skeletal muscle mass compared with adults.

To provide a robust answer to the question of whether RIPC can attenuate perioperative myocardial injury in young children undergoing surgery, we conducted a randomized, prospective, double-blind trial comparing state-of-the-art bilateral preconditioning with a sham control in children with the 2 most common congenital heart defects requiring surgery and investigated the impact of RIPC on myocardial metabolism during cardioplegic arrest.

## Materials and Methods

### Study Design

**Figure 1:**
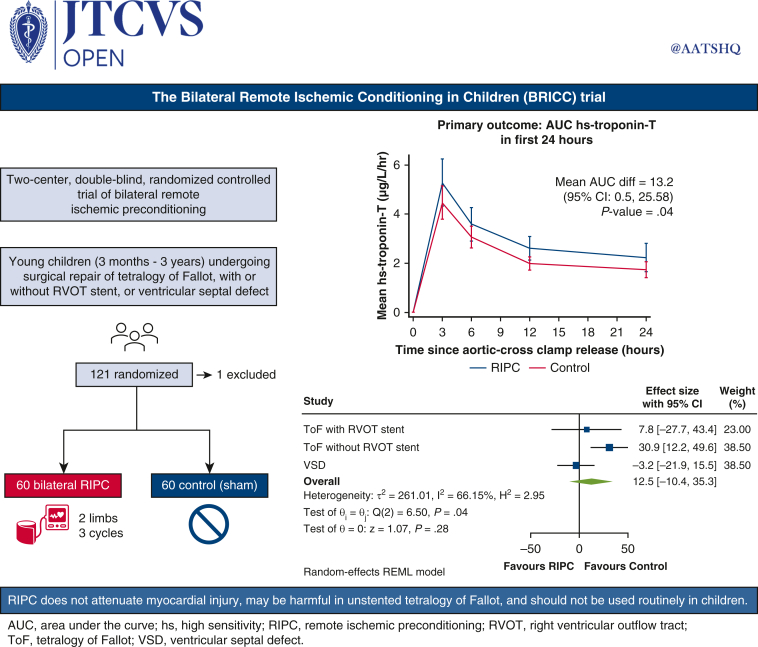
The Bilateral Remote Ischemic Conditioning in Children trial. A 2-center, double-blind, randomized controlled trial in which 120 young children with the 2 most common congenital heart defects requiring surgery were randomized to bilateral RIPC or sham intervention. AUC for hs-troponin-T in the first 24 hours was higher in the preconditioning group than in controls (*P* = .04), and subgroup analysis suggested a differential effect by underlying defect (p_interaction =_ .04). Bilateral RIPC does not attenuate myocardial injury during surgery in young children, with evidence of potential harm in unstented TOF, and its routine use cannot be recommended. *AUC*, Area under the curve; *CI*, confidence interval; *RVOT*, right ventricular outflow tract; *RIPC*, remote ischemic preconditioning; *ToF*, tetralogy of Fallot; *VSD*, ventricular septal defect; *hs*, high sensitivity.

The Bilateral Remote Ischemic Conditioning in Children trial was a double-blind, prospective, parallel group, randomized controlled trial in young children undergoing elective cardiac surgery at 2 centers in the United Kingdom: Birmingham Children's Hospital and Leeds Children's Hospital (Figure 1). The study was approved by the West Midlands-Solihull NHS Research Ethics Committee (16/WM/0309, August 5, 2016). The published trial protocol provides a detailed description of the trial and methods, including patient and public involvement.^17^

### Patients

All infants and young children, aged 3 months to 3 years at the time of surgery, undergoing complete repair of tetralogy of Fallot (TOF) or surgical closure of a ventricular septal defect (VSD), with or without concomitant atrial septal defect closure or pulmonary artery repair/augmentation, were eligible. Children were excluded if they required an additional procedure (other than atrial septal defect closure or pulmonary artery repair/augmentation); had significant airway or parenchymal lung disease, bleeding disorder, or a recent ischemic event; had undergone previous cardiac surgery with cardioplegic arrest; required emergency surgery; or their parents declined to give consent. As in previous trials,^6^^,^^12^ those with a known major chromosomal defect were also initially excluded, but this was amended during the trial because there was no biological reason for exclusion. Eligible patients were identified from multidisciplinary team meetings, clinics, or surgical waiting lists. A parent information sheet was provided, in person or via post, and parental written informed consent for publication obtained by a consultant surgeon before enrollment.

### Randomization and Blinding

On the day of surgery, participants were randomized (1:1) to receive RIPC or a sham procedure (control) using a secure online randomization system with a minimization algorithm incorporating (1) congenital heart defect, (2) presence of a right ventricular outflow tract (RVOT) stent in those with TOF, and (3) surgical center.

The trial intervention was delivered by an independent healthcare professional trained and competent in its delivery according to a standard operating procedure, who also performed the randomization and was not involved in postoperative care. Blinding was maintained by covering the child with a surgical drape throughout cuff application, intervention, and removal. The research nurse and clinical teams involved in the child's care were blinded to group allocation throughout the trial.

### Procedures

After induction of anesthesia but before sternotomy, the treatment group received RIPC in both lower limbs simultaneously using the PTSii digital tourniquet system (Delfi Medical Innovations) inflated to 50 mm Hg or greater above systolic pressure for 3 cycles of 5 minutes ischemia and 5 minutes reperfusion^18^; if 1 lower limb was unavailable, the cuff was placed on the upper arm. Continual loss of arterial flow during ischemia was confirmed by concealed distal pulse oximetry.^10^ Once the intervention had begun, each cuff was kept on the same limb to ensure repeated doses of ischemia-reperfusion to the same muscle mass. In the control group, cuffs were applied to a plastic tubing dummy limb placed between the participant's legs and 3 sham inflation-deflation cycles performed, covered by a drape before, during, and after the sham intervention to maintain blinding. Adherence to intervention was defined as receiving the allocated intervention, with documented loss of arterial flow during each ischemic phase in the RIPC group.

All other aspects of anesthesia, surgery, perfusion, and postoperative care were at the sole discretion of the blinded clinical team, except for propofol, which was not used for induction or maintenance of anesthesia, with isoflurane as the preferred volatile anesthetic.^19^ St Thomas' cardioplegia was used at both sites and delivered according to local practice. Myocardial reperfusion on first release of the aortic crossclamp was considered as time zero for postoperative events. Blood was drawn before sternotomy and at 3, 6, 12, and 24 hours after reperfusion, and plasma high-sensitivity (hs) troponin-T concentrations were quantified in batches using the fifth-generation Elecsys Tn-T HS assay (Roche) at an approved core laboratory.

Right atrial biopsies were obtained soon after aortic crossclamping (onset ischemia) and just before its release (late ischemia) for metabolic phenotyping. Briefly, tissue extracts were analyzed using ultra high-performance liquid chromatography-mass spectrometry. Two complementary assays were applied: HILIC assay for water-soluble metabolites and C_18_ reversed-phase assay for lipids. The impact of RIPC on myocardial metabolism was assessed through robust statistical analysis using correction for multiple testing and pathway enrichment analysis (Supplementary Methods).

### Outcomes

The primary outcome was area under the curve (AUC) for plasma hs-troponin-T in the first 24 hours after aortic crossclamp release (reperfusion) as a biomarker of myocardial injury. Secondary outcomes were peak hs-troponin-T in the first 12 hours; total vasoactive inotrope score in the first 12 hours^20^; arterial lactate and central venous oxygen saturations in the first 12 hours; length of postoperative stay in intensive care unit (ICU); and length of postoperative stay in hospital. Cardiac index was measured using ICON (Osypka Medical) as an exploratory outcome in Birmingham only (Supplemental Methods).

The following serious adverse events were reported to the sponsor within 48 hours of identification: death; extracorporeal life support; major neurological event; and further surgery or catheter intervention in the early postoperative period. Follow-up was until discharge from hospital or 30 days, whichever was sooner.

### Sample Size

We hypothesized that RIPC would reduce AUC for hs-troponin-T in the first 24 hours compared with controls, but that exposure to chronic hypoxemia may impact this reduction. Based on limited published data using a standard (fourth-generation) troponin assay, the proposed sample size was sufficient to detect a 35% reduction in postoperative AUC troponin, assuming a mean release equivalent to 350 μg/L/h in the control group compared with 228 μg/L/h in the RIPC group (extrapolated from the similarly mixed cohort of cyanotic and acyanotic children^6^), with a variability of 220 μg/L/h^9^ A sample size of at least 52 children per treatment group was needed to have a power of 80% and a significance level of .05 (2-sided). We aimed to recruit up to 120 children to allow for dropouts, randomized in a 1:1 ratio between RIPC and control.

### Statistical Analysis

Primary analysis of the primary and secondary outcome measures was performed according to the intention-to-treat principle. Analyses were undertaken using SAS v9.4. The primary comparison compared the RIPC group with the control group, and all estimates of differences are presented with 95% CIs.

To calculate the primary outcome, AUC for hs-troponin-T in the first 24 hours, data were collected at baseline (presternotomy) and at 3, 6, 12, and 24 hours after reperfusion. The AUC was calculated for each participant using the trapezoidal method and compared between groups using a linear regression model, adjusting for the minimization variables (congenital heart defect, center) and baseline troponin. Missing baseline troponin values were imputed using the median value of the participant's randomized group and type of defect, whereas any missing postoperative value led to exclusion from the primary analysis. Participants who received their randomized treatment, including those with incomplete postoperative troponin data, were included in the per-protocol analysis. Subgroup analyses were performed for the primary outcome only to assess whether the treatment effect differed by preoperative oxygen saturations (cyanotic < 90% or acyanotic ≥ 90%); congenital heart defect (TOF with RVOT stent, TOF without RVOT stent, or VSD); and age (<1 year or ≥1 year).

For the secondary outcomes, continuous data items were analyzed using a linear regression model. Continuous outcomes measured across more than 3 time points were analyzed using mixed-effect repeated-measures models. Time to event outcomes were analyzed using a Cox regression model. *P* values are reported from 2-sided tests. The statistical analysis plan was agreed and signed off before database lock, and the Chief Investigator and trial statisticians had access to the final dataset.

The trial was prospectively registered (ISRCTN12923441) before recruiting the first patient. An independent Data Monitoring Committee (see Acknowledgments) met at regular intervals during recruitment to review efficacy and safety data. As Chief Investigator, the first author takes responsibility for the integrity of this report. All authors have read and agree to the manuscript as written. The funder had no role in study design, data collection, analysis, interpretation, or reporting.

## Results

**Figure E1:**
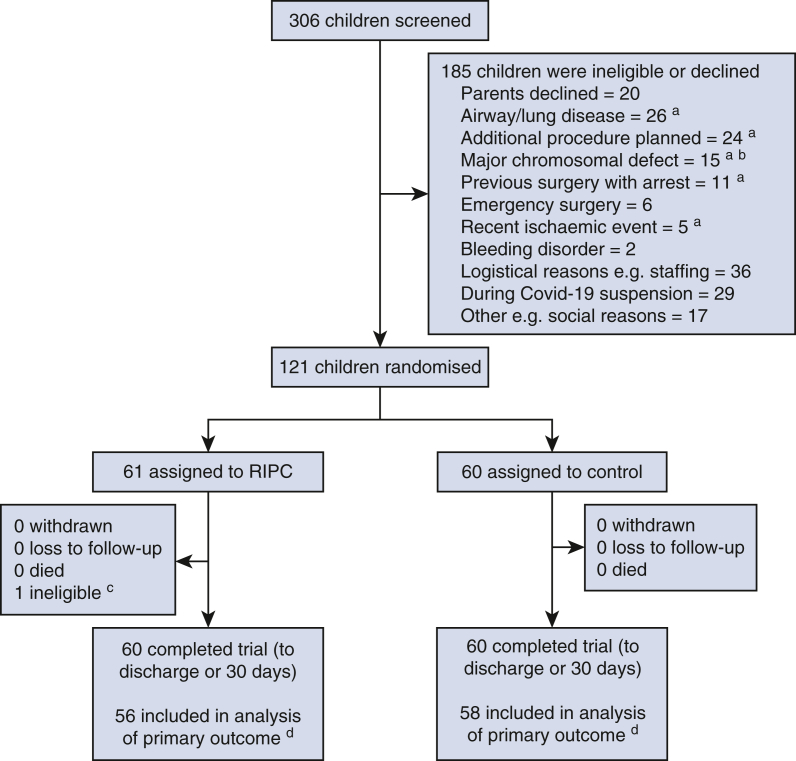
CONSORT flow diagram. In this trial, 120 young children undergoing surgery for congenital heart disease were randomized to bilateral RIPC or sham intervention and completed follow-up. ^a^Patients counted in multiple categories due to concomitant reasons for exclusion: 191 reasons in 185 patients. ^b^Before change to exclusion criteria to allow recruitment. ^c^Participant randomized in error, later deemed ineligible, did not undergo surgery; therefore, outcome data were not available. Excluded postrandomization, before receipt of any intervention and not included in subsequent analyses. ^d^With complete primary outcome data available. *RIPC*, Remote ischemic preconditioning.

Between October 24, 2016, and December 8, 2020, 306 children were screened, of whom 223 (72.9%) met the trial eligibility criteria and of these, 82 (36.8%) were excluded for logistical or other reasons; 20 (14.2%) parents of otherwise eligible children declined consent. The CONSORT flow diagram is shown in Figure E1. Recruitment was paused between March 13, 2020, and June 29, 2020, due to the impact of the Coronavirus Disease 2019 pandemic on the National Health Service (Supplementary Methods). A total of 121 infants/young children were randomized: 61 to RIPC and 60 to control. One child in the RIPC group was later found to be ineligible and did not proceed to surgery so was excluded from the analysis, leaving 60 participants in each group who underwent surgery and completed follow-up. Baseline characteristics were similar between groups (Table 1), and operative data are shown in Table 2. Adherence to treatment allocation was achieved in 116 (96.7%) participants, 56 (93%) in the RIPC group, who received 3 confirmed cycles of limb ischemia-reperfusion, and 60 (100%) in the control group (Supplementary Results). There was 1 (<1%) protocol deviation with propofol inadvertently used for induction of anesthesia in a participant in the RIPC arm.

**Table 1:** Baseline participant characteristics by treatment group and overall

Participant characteristics	RIPC (n = 60)	Control (n = 60)	Overall (n = 120)
Age, mo	7.0 [4.5-12.0]	8.0 [4.5-12.5]	7.0 [4.5-12.0]
Weight, kg	7.2 [6.0-8.5]	7.1 [6.3-8.5]	7.1 [6.1-8.5]
Male/female (n, n)	42, 18	36, 24	78, 42
Ethnic group, n (%)			
White	41 (68%)	44 (73%)	85 (71%)
Asian	11 (18%)	9 (15%)	20 (17%)
Black	3 (5%)	5 (8%)	8 (7%)
Mixed	4 (7%)	2 (3%)	6 (5%)
Other	1 (2%)	0 (-)	1 (<1%)
Congenital heart defect, n (%)∗			
TOF without RVOT stent	27 (45%)	27 (45%)	54 (45%)
TOF with RVOT stent	7 (12%)	7 (12%)	14 (12%)
VSD	26 (43%)	26 (43%)	52 (43%)
Preoperative cyanosis, n (%)†	20 (33%)	21 (35%)	41 (34%)
Preoperative O_2_ saturations, %			
TOF without RVOT stent	89 [86-97]	87 [84-97]	88 [86-97]
TOF with RVOT stent	84 [76-92]	83 [81-90]	83.5 [81-90]
VSD	98 [97-99]	98 [97-100]	98 [97-99.5]
Preoperative hematocrit, %			
TOF without RVOT stent	39.9 [37.2-42.2]	38.7 [37.2-42.7]	39.0 [37.2-42.2]
TOF with RVOT stent	37.8 [34.4-41.2]	39.7 [39.3-44.5]	39.5 [37.8-41.2]
VSD	35.0 [31.6-37.0]	32.9 [30.9-34.2]	33.7 [31.3-36.1]
Recent spelling, n (%)	15 (25%)	14 (23%)	29 (24%)
Known genetic anomaly, n (%)	8 (13%)	12 (20%)	20 (17%)
Trisomy 21	4 (7%)	7 (12%)	11 (9%)
Other chromosomal	4 (7%)	5 (8%)	9 (8%)
Previous cardiovascular surgery or catheter intervention, n (%)	12 (20%)	12 (20%)	24 (20%)
RVOT stent	7 (12%)	7 (12%)	14 (12%)
Pulmonary artery band	4 (7%)	3 (5%)	7 (6%)
Blalock-Thomas-Taussig shunt	0 (-)	1 (2%)	1 (<1%)
Other	1 (2%)	1 (2%)	2 (2%)
Pre-operative medication			
Beta-blocker	12 (20%)	13 (22%)	25 (21%)
Diuretic	16 (27%)	15 (25%)	31 (26%)

Data are median [IQR] when appropriate. *RIPC*, Remote ischemic preconditioning; *TOF*, tetralogy of Fallot; *RVOT*, right ventricular outflow tract; *VSD*, ventricular septal defect.

∗ Minimization variable.

† Preoperative cyanosis defined as resting oxygen saturations less than 90% on room air.

**Table 2:** Operative data by treatment group

Variables	RIPC (n = 60)	Control (n = 60)
Anesthesia		
Volatile anesthetic agent: isoflurane	56 (93%)	55 (92%)
Volatile anesthetic agent: sevoflurane	4 (7%)	5 (8%)
MAC volatile anesthetic pre-CPB, %	0.7 [0.6-0.9]	0.8 [0.7-1.0]
Time from start of intervention to aortic XC, min	93 [81-105]	92 [77-103]
In TOF repair	(n = 34)	(n = 34)
RVOT muscle resected	34 (100%)	33 (97%)
RVOT stent removed∗	6 (86%)	7 (100%)
RVOT/TA/PA patch used	32 (94%)	33 (97%)
RV-PA conduit used	2 (6%)	1 (3%)
In isolated VSD closure	(n = 26)	(n = 26)
RVOT muscle resected	5 (19%)	3 (12%)
VSD closed	60 (100%)	59 (98%)†
Total CPB time, min	91.0 [67.0-114.0]	87.5 [70.0-106.5]
Repeat CPB required	7 (12%)	5 (8%)
Total aortic XC time, min	64.0 [45.0-84.5]	58.5 [44.5-74.0]
Repeat aortic XC required	6 (10%)	5 (8%)
Type of cardioplegia used		
Cold blood	54 (90%)	53 (88%)
Cold crystalloid	6 (10%)	6 (10%)
Warm blood	0	1 (2%)
No. of cardioplegia doses	2 [2-3]	2 [2-3]
Total cardioplegia volume, mL	360 [272-450]	369 [294-450]
Postoperative recovery		
Drain loss at 12 h, mL	55.0 [45.0-87.5]	79.5 [40.0-107.5]
Blood transfusion post-CPB in first 12 h	13 (22%)	11 (18%)
Volume blood transfused in first 12 h, mL/kg in those transfused	75 [55-100]	50 [30-80]
Hemoglobin at 12 h, g/dL	119 [111-134]	119 [107-130]
Time to extubation, h	6.3 [3.7-15.2]	6.9 [3.6-13.3]

Data are median [IQR] when appropriate. *RIPC*, Remote ischemic preconditioning; *MAC*, minimum alveolar concentration; *CPB*, cardiopulmonary bypass; *XC*, crossclamp; *TOF*, tetralogy of Fallot; *RVOT*, right ventricular outflow tract; *TA*, transannular; *PA*, pulmonary artery; *RV-PA*, right ventricle to pulmonary artery; *VSD*, ventricular septal defect.

∗ Of the 7 participants in each treatment arm with TOF and an RVOT stent.

† Small infant with TOF and bilateral superior vena cavae in whom an RV-PA conduit was placed but complete repair was abandoned.

### Primary Outcome

**Figure 2:**
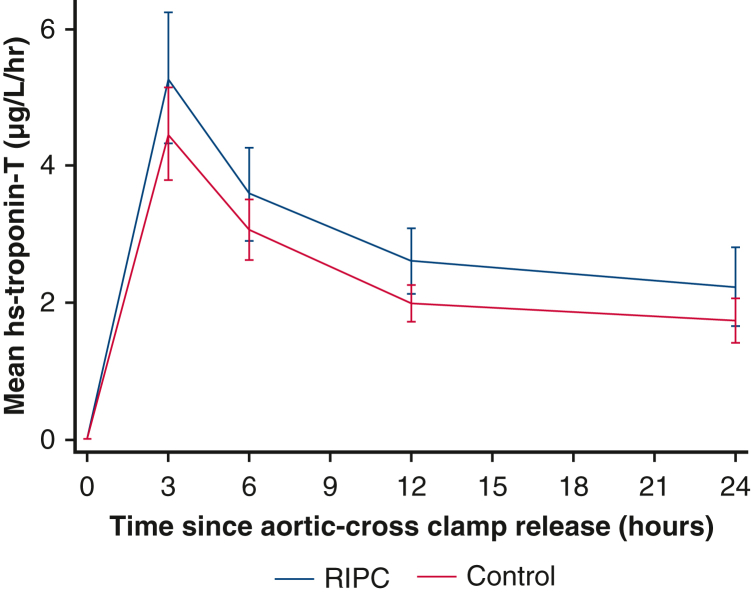
Mean hs-troponin-T release in the first 24 hours after surgery by treatment group. Mean AUC for hs-troponin-T was higher (ie, worse) in the RIPC group (70.0 ± 50.9 μg/L/h) compared with control (55.6 ± 30.16 μg/L/h), mean difference 13.2 μg/L/h (95% CI, 0.5-25.8; *P* = .04). *RIPC*, Remote ischemic preconditioning.

**Figure 3:**
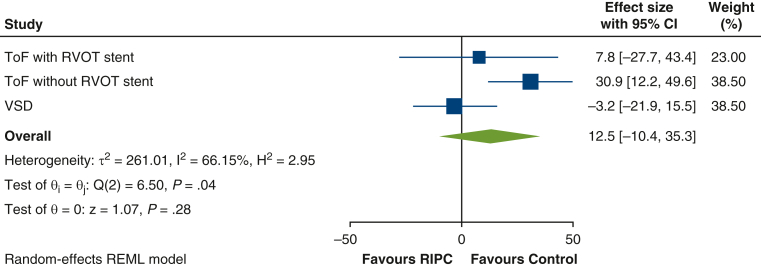
Forest plot of the primary outcome by congenital heart defect group. The overall estimate is the weighted average of the observed treatment effects for each congenital heart defect group, estimated using random effects to allow for variation between groups. *CI*, Confidence interval; *TOF*, tetralogy of Fallot; *RVOT*, right ventricular outflow tract; *VSD*, ventricular septal defect; *RIPC*, remote ischemic preconditioning.

Complete postoperative troponin data were available for 114 children (56 RIPC, 58 control) who were included in the primary analysis. Overall, the mean AUC for hs-troponin-T was higher (ie, worse) in the RIPC group (70.0 ± 50.9 μg/L/h) compared with control (55.6 ± 30.16 μg/L/h) (mean difference 13.2 μg/L/h; 95% CI, 0.5-25.8; *P* = .04) (Figure 2 and Table E1). The per-protocol analysis supported the primary analysis (mean difference 15.5; 95% CI, 2.6-28.3; *P* = .02). Prespecified subgroup analyses did not show a differential treatment effect in cyanotic and acyanotic children (interaction *P* value = .25). However, for age and congenital heart defect, there was some evidence of a possible interaction: age less than 1 year, mean difference 20.8 (95% CI, 6.1-35.4) versus 1 year or more, mean difference −6.7 (95% CI, −30.6 to 17.3) (interaction *P* value = .06); congenital heart defect: unstented TOF, mean difference 30.9 (95% CI, 12.2-49.6); stented TOF, mean difference 7.8 (95% CI, −27.7, 43.4); VSD, mean difference −3.2 (95% CI, −22.0 to 15.5) (interaction *P* value = .04) (Figure 3, Table 3, Tables E2 and E3).

**Table 3:** Predefined subgroup analyses for the primary outcome, area under the curve for plasma high-sensitivity troponin-T release in the first 24 hours after reperfusion

Subgroups	RIPC mean (SD, n)	Control mean (SD, n)	Interaction *P* value	Mean difference (95% CI)∗
Congenital heart defect				
TOF with RVOT stent	74.4 (23.1, 7)	67.8 (41.7, 7)	.04	7.8 (−27.7 to 43.4)
TOF without RVOT stent	102.5 (57.5, 25)	69.3 (25.3, 25)		30.9 (12.2-49.6)
VSD	35.0 (13.5, 24)	39.3 (23.1, 26)		−3.2 (−22.0 to 15.5)

Subgroups	RIPC mean (SD, n)	Control mean (SD, n)	Interaction *P* value	Mean difference (95% CI)†
Preoperative oxygen saturations				
<90% (cyanotic)	98.5 (58.6, 19)	73.6 (34.1, 20)	.25	23.5 (2.0-45.1)
≥90% (acyanotic)	55.4 (40.0, 37)	46.2 (23.1, 38)		7.8 (−7.9 to 23.4)
Age				
<1 y	79.2 (55.6, 41)	56.3 (26.5, 42)	.06	20.8 (6.1-35.4)
≥1 y	44.9 (21.2, 15)	54.0 (39.0, 16)		−6.7 (−30.6 to 17.3)

*RIPC*, Remote ischemic preconditioning; *SD*, standard deviation; *CI*, confidence interval; *TOF*, tetralogy of Fallot; *RVOT*, right ventricular outflow tract; *VSD*, ventricular septal defect.

∗ Mean difference (RIPC – Control) calculated using linear regression model, adjusting for baseline troponin and center.

† baseline troponin, congenital heart defect and center. A negative difference favors the RIPC group.

### Secondary Outcomes and Adverse Events

**Figure E2:**
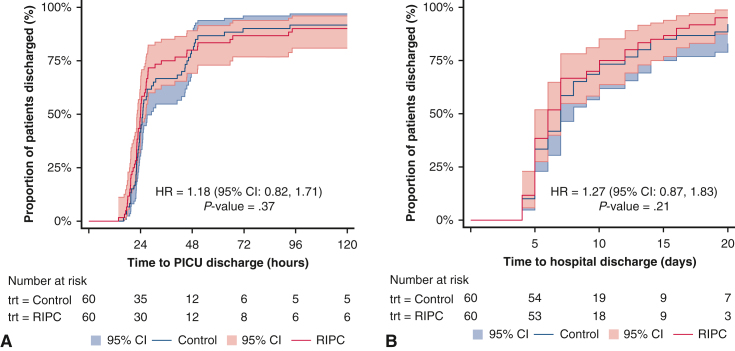
Kaplan–Meier plots of time to discharge from (A) pediatric ICU, by treatment group with data censored at 336 hours (14 days), and (B) hospital, by treatment group with data censored at 30 days. Pediatric ICU. 95% CI. *HR*, Hazard ratio; *CI*, confidence interval; *RIPC*, remote ischemic preconditioning; *PICU*, pediatric intensive care unit; *trt*, treatment group.

There were no differences between groups in any of the secondary outcome measures (Table 4 and Figure E2) or the exploratory outcome (Table E4). No immediate limb complications were observed, and there were no differences in adverse events (Table 5). There were 11 serious adverse events in 9 participants: 4 in the RIPC group and 5 in the control group (Supplementary Results); all were assessed to be unrelated to the trial intervention.

**Table 4:** Secondary outcomes by treatment group

Outcomes	RIPC mean (SD, n)	Control mean (SD, n)	Point estimate (95% CI), *P* value
Peak Hs-troponin-T in first 12 h (μg/L)	5.42 (3.64, 57)	4.51 (2.54, 58)	0.85 (−0.09 to 1.80), .08∗
Vasoactive inotrope score in first 12 h	61.6 (39.0, 60)	53.6 (32.3, 60)	8.1 (−4.1 to 20.3), .19†
Arterial lactate (mmol/L)			
Baseline	1.06 (0.29, 58)	1.10 (0.56, 59)	0.04 (−0.12 to 0.20), .60‡
3 h	1.69 (0.61, 60)	1.61 (0.65, 59)	
6 h	1.73 (0.75, 57)	1.73 (0.69, 58)	
9 h	1.50 (0.71, 55)	1.62 (1.25, 55)	
12 h	1.45 (0.49, 55)	1.47 (0.82, 55)	
Central venous oxygen saturations (%)			
3 h	64.2 (10.2, 56)	65.4 (8.7, 55)	−1.10 (−3.85 to 1.66), .43‡
6 h	61.9 (12.8, 55)	63.9 (9.2, 57)	
9 h	60.7 (10.8, 50)	62.2 (8.3, 50)	
12 h	61.4 (7.7, 51)	61.8 (10.4, 57)	

Outcomes	RIPC median (IQR, n)	Control median (IQR, n)	Point estimate (95% CI), *P* value§
Time to ICU discharge (h)	23.92 (20.55-33.58, 59)	24.95 (22.00-45.57, 59)	1.18 (0.82-1.71), .37
Time to ICU discharge (h)¦	23.96 (20.83-36.78, 60)	25.00 (22.02-45.91, 60)	1.20 (0.84-1.73), .32
Time to hospital discharge (d)	6.0 (5.0-10.0, 59)	7.0 (5.0-10.0, 57)	1.27 (0.88-1.83), 0.21
Time to hospital discharge (d)‖	6.0 (5.0-11.0, 60)	7.0 (5.0-12.0, 60)	1.27 (0.88-1.84), 0.20

*RIPC*, Remote ischemic preconditioning; *SD*, standard deviation; *CI*, confidence interval; *hs*, High-sensitivity; *IQR*, interquartile range; *ICU*, intensive care unit.

∗ Postoperative time points from first release of the aortic crossclamp. Mean difference (RIPC – Control) calculated using linear regression model, adjusting for baseline troponin, congenital heart defect and center.

† congenital heart defect and center; a negative difference favors the RIPC group.

‡ Average mean difference over each time point (RIPC – Control) calculated using a mixed linear regression model adjusting for baseline value of the measure (if available), heart condition and center. For arterial lactate, a negative difference favors the RIPC group; for central venous oxygen saturations, a positive difference favors the RIPC group.

§ Hazard ratio (RIPC/Control) calculated using a Cox proportional hazards regression model, adjusted for center and congenital heart defect; values greater than 1 favor RIPC.

‖ Using uncensored data.

**Table 5:** Adverse events and serious adverse events by treatment group

Adverse events	RIPC (n = 60)	Control (n = 60)
Cardiac complication∗	7 (12%)	6 (10%)
Cardiac arrest	0	1 (2%)
ECLS required†	1 (2%)	1 (2%)
Postoperative complete heart block	1 (2%)	1 (2%)
Reoperation required†	4 (7%)	4 (7%)‡
Pericardial collection/bleeding	2 (3%)	1 (2%)
Residual lesions	1 (2%)	2 (3%)
Epicardial pacemaker fitted	1 (2%)	1 (2%)
Chest left open	4 (7%)	2 (3%)
Post-CPB bleeding	1 (2%)	0
Prolonged CPB time	3 (5%)	1 (2%)
Other	0	1 (2%)
Neurological event†	0	0
Renal support, CVVH or PD	1 (2%)	2 (3%)
Peak creatinine, μmol/L	25 [20-31]	24 [21-30]
Respiratory event	2 (3%)	2 (3%)
Reintubated	0	1 (2%)
Infection – systemic	4 (7%)	1 (2%)
Infection – surgical site	1 (2%)	0 (0%)
Limb complication	0	0
Other adverse event	0	0
Death after surgery†	0	0

Data are median [IQR] when appropriate. *RIPC*, Remote ischemic preconditioning; *ECLS*, extracorporeal life support; *CPB*, cardiopulmonary bypass; *CVVH*, continuous veno-venous hemofiltration; *PD*, peritoneal dialysis.

∗ Two participants experienced 3 adverse events each, and 2 participants experienced 2 adverse events each. In total, 19 adverse events occurred in 13 participants.

† Serious adverse event requiring expedited reporting.

‡ One participant underwent 2 reoperations to fit a temporary then permanent epicardial pacemaker; in total, 5 reoperations occurred in 4 participants.

### Metabolic Phenotyping

Right atrial biopsies were collected at the onset of ischemia (median 4 minutes after crossclamping) and at the end of ischemia (median 53 minutes after crossclamping), and analysis was performed in 40 participants, 20 in each group. With correction for multiple testing (q < 0.05), in both early and late ischemia, there were no differences in myocardial tissue metabolites or enrichment of metabolic pathways between participants receiving RIPC or sham intervention.

## Discussion

We found that adequately delivered, perioperative, bilateral RIPC, compared with sham inflation-deflation cycles, did not reduce myocardial injury in children undergoing elective surgery for common congenital heart defects. Troponin was slightly higher in patients randomized to RIPC; this effect was increased in the per protocol analysis and was mainly due to greater postoperative troponin in children with unstented TOF. This is the first trial to directly compare outcomes of RIPC in children with cyanotic and acyanotic congenital heart defects undergoing surgery at a similar age,^16^ and our findings suggest that bilateral RIPC may exacerbate myocardial ischemia-reperfusion injury in children with TOF. RIPC did not alter postoperative inotropic support, length of ICU or hospital stay, or postoperative complications. The immediate clinical implication of our results is to avoid RIPC during pediatric cardiac surgery.

**Figure E3:**
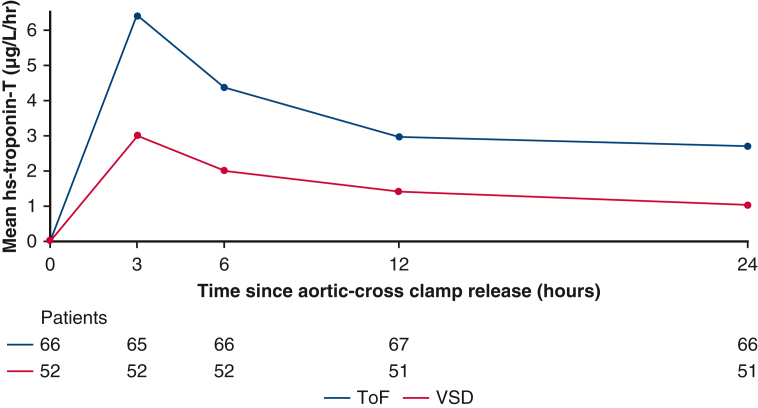
Mean hs-troponin-T release in the first 24 hours by congenital heart defect. *TOF*, Tetralogy of Fallot; *VSD*, ventricular septal defect.

Overall AUC hs-troponin-T was higher in patients with TOF compared with those with an isolated VSD (Figure E3), reflecting a longer ischemic time and more frequent RVOT muscle resection. On subgroup analysis, we found no difference in troponin related to preoperative oxygen saturations above or below 90%, but there was some evidence of an interaction with age (*P* = .06), suggestive of greater myocardial injury in those aged less than 1 year who received RIPC, independent of defect type.

The promise of this simple, low-risk, inexpensive, and readily available intervention as an adjunct to current methods for myocardial protection has prompted numerous trials in adults^21^, ^22^, ^23^, ^24^, ^25^, ^26^ and children^6^, ^7^, ^8^, ^9^, ^10^, ^11^, ^12^, ^13^, ^14^ but with mixed results. Two large multicenter trials in adults failed to show benefit in composite cardiovascular end points or troponin release,^25^^,^^26^ but both studies were criticized for using propofol after it had been shown to interfere with the preconditioning pathway.^15^ In a 2017 meta-analysis that included 793 children from 9 trials, Tan and colleagues^27^ determined that RIPC has a cardioprotective effect, with reduced troponin at 6 hours, lower inotrope scores in the first 12 hours, and reduced postoperative ICU stay; however, they were unable to include the largest trial (n = 299) in most analyses due to a lack of suitable published data. Therefore, our trial makes an important contribution to the literature, raising major doubts about the use of RIPC in pediatric cardiac surgery.

Cheung and colleagues^6^ first demonstrated reductions in troponin and inotropic requirements with RIPC in a heterogeneous cohort of 37 children, most of whom had TOF or VSD, a similar population to our trial. Several small studies found improved myocardial protection, with reduced early troponin in young children undergoing VSD closure,^7^^,^^8^ whereas others found no benefit in neonates or infants undergoing other cardiac operations.^9^, ^10^, ^11^^,^^14^ In the largest pediatric trial of RIPC to date, McCrindle and colleagues^12^ found no benefit in clinical outcomes, physiological biomarkers, or subgroup analyses in a mixed cohort of 299 children aged 0 to 17 years and proposed that better than expected outcomes in the control group, heterogeneity of underlying conditions, and use of propofol may have affected their findings. Failure to elicit a stimulus may have been a key factor; manual inflation of the cuff to just 15 mm Hg above systolic pressure may have led to periods of subclinical reperfusion and the abolition of any preconditioning response. However, our findings support those of McCrindle and colleagues that RIPC has little or no effect on clinical end points, and therefore its role as a protective strategy in children is limited.

A double-blind, randomized trial of 112 children undergoing complete repair of TOF in Wuhan, China, published in 2018 found a reduced duration of ICU stay and lower AUC for troponin-T in the first 24 hours with RIPC versus control.^13^ Although patients underwent surgery at a similar age (11 months) as that in our trial, they were primarily acyanotic with mean preoperative oxygen saturations of 97% to 98% (SD, ±3%-4%), which is markedly different to our unstented TOF group (median, 88%; IQR, 86-97) in whom recent hypercyanotic spells were common. The authors also did not mention any prerepair interventions for severe cyanosis, either Blalock-Thomas-Taussig shunt or RVOT stenting. These factors suggest that the pattern of TOF in their population is different than that seen in Europe and North America,^28^ or elsewhere in China.^29^ This may be due to the higher incidence of a fibrous rather than muscular outlet septum in TOF in parts of East Asia, reducing the extent of RVOT obstruction and cyanosis,^30^ and explain the divergence of findings.

The mechanism underlying RIPC and its potential interaction with chronic cyanosis in ischemia-reperfusion injury associated with cardioplegic arrest is not clearly understood. RIPC has been shown to induce regulatory phosphorylation of key intracellular proteins involved in pro-survival metabolic signaling.^31^^,^^32^ Pepe and colleagues^10^ evaluated the effect of RIPC on phosphorylated protein signaling in cyanotic children with TOF undergoing surgical repair; they found that signaling pathways were upregulated in resected RVOT muscle from both groups, supporting the hypothesis that the children may already have been preconditioned by exposure to chronic hypoxemia since birth, preempting any potential benefit from RIPC. In the same cohort, Hepponstall and colleagues^33^ reported postoperative plasma proteomic changes in the RIPC group, with higher expression of proteins involved in metabolism, hemostasis, immunity, and inflammation that may be involved in RIPC-mediated cellular protection. In experimental models of ischemia-reperfusion, accumulation of succinate is a key metabolic signature of ischemia and drives the generation of mitochondrial reactive oxygen species during reperfusion, leading to cellular injury.^34^ Therefore, we postulated that RIPC may impact the myocardial metabolic phenotype during ischemia; however, our analysis of right atrial samples found no significant differences in metabolites or metabolic pathways between the RIPC and control arms. These novel findings suggest that downstream myocardial metabolism does not play a major role in mediating the effects of RIPC in children undergoing cardioplegic arrest.

In this trial, we addressed the methodological concerns raised over previous trials. A pressure-controlled digital tourniquet system was used, set to 50 mm Hg or greater above systolic pressure to avoid subclinical reperfusion during the ischemic phase of each cycle,^12^ with loss of arterial flow confirmed by distal pulse oximetry. A more intensive 2-cuff technique was used,^24^ applying a concurrent stimulus to both lower limbs to compensate for the lower skeletal muscle mass. We avoided propofol anesthesia, which has been shown to attenuate the effects of RIPC^15^^,^^19^ and excluded infants aged less than 3 months in whom the immature myocardium may be less responsive to RIPC.^11^ This trial is the first multicenter cardiac surgical trial in children in the United Kingdom and demonstrates the value of collaboration to achieve recruitment targets in this challenging field.

### Study Strengths and Limitations

The randomized, double-blind design, robust and verified delivery of bilateral RIPC, and quantification of a validated biomarker outcome are key strengths of our study. The results are consistent across analyses, including sensitivity analyses and comparison of secondary outcomes. The sample size calculation was based on analysis of the primary outcome in all participants, and therefore subgroup analyses should be seen as exploratory. It was also based on published studies using standard (fourth-generation) troponin assays, whereas we used an hs (fifth-generation) assay, which is now the preferred biomarker for determining myocardial injury.^35^ Although troponin isoforms may be released from injured skeletal muscle, we found no difference between the RIPC and control groups in patients with a VSD, reassuring that the bilateral RIPC stimulus did not cause significant peripheral troponin-T release. Resection of right ventricular infundibulum or removal of an RVOT stent is likely to have increased troponin release, narrowing the effect of RIPC and predisposing to a type II error; despite this, RIPC remained associated with a greater AUC in patients with TOF. We defined preoperative cyanosis as resting oxygen saturations in room air of less than 90%, but TOF is a dynamic condition in which the right-to-left shunt and saturations may fluctuate, weakening the use of a single measurement to determine dichotomous groups; this may have partly accounted for the differential effects seen by congenital heart defect versus preoperative oxygen saturations.

## Conclusions

Bilateral RIPC does not improve cardioprotection in young children undergoing operative repair of common congenital heart defects and may be harmful in those with unstented TOF. Therefore, the routine use of RIPC cannot be recommended, and alternative methods to improve myocardial protection and outcomes of surgery for congenital heart disease should be sought.

## Trial Management Committee

Nigel E. Drury (Chief Investigator), Timothy J. Jones (Principal Investigator, Birmingham), Carin van Doorn (Principal Investigator, Leeds), Paulus Kirchhof, Rehana Bi, Alicia Gill, Natalie J. Ives, Melanie Madhani, James Montgomerie, Kevin P. Morris, Collette Spencer, John Stickley, and Rebecca L. Woolley.

## Clinical Teams

Surgeons – Birmingham: Timothy Jones, David Barron, Phil Botha, Natasha Khan, and Leeds: Carin van Doorn, Osama Jaber, Imre Kassai, Guiseppe Pelella. Anesthetists – Birmingham: Dr James Montgomerie, Dr Edmund Carver, Dr Alistair Cranston, Dr Fraser Harban, Dr Vasco Laginha Rolo, Dr Ritchie Marcus, Dr Tony Moriarty, Dr Raju Reddy, Dr Susanna Ritchie-McLean, Dr Monica Stokes, Dr Ayngara Thillaivasan, and Leeds: Dr Nandlal Bhatia, Dr Carol Bodlani, Dr Wendy Lim, Dr Joe Mellor, Dr Jutta Scheffczik.

## Data Availability

Requests for access to data should be addressed to the Chief Investigator. Individual participant data collected during the trial (including the data dictionary) will be available, after deidentification, when the article has been published with no end date. All proposals requesting data access must specify how the data will be used, and all proposals will need the approval of the Trial Management Committee before data release.

## Conflict of Interest Statement

The authors reported no conflicts of interest.

The *Journal* policy requires editors and reviewers to disclose conflicts of interest and to decline handling or reviewing manuscripts for which they may have a conflict of interest. The editors and reviewers of this article have no conflicts of interest.

## Figures and Tables

**Table E1:** Mean plasma high-sensitivity troponin-T at each time point by treatment group

Time point	Plasma hs-troponin-T (μg/L), mean (SD, n)	
	RIPC group	Control group
Baseline	0.02 (0.02, 59)	0.02 (0.02, 59)
3 h	5.31 (3.66, 59)	4.49 (2.56, 58)
6 h	3.60 (2.65, 59)	3.08 (1.68, 59)
12 h	2.62 (1.85, 59)	2.00 (1.02, 59)
24 h	2.24 (2.19, 58)	1.75 (1.23, 59)

Postoperative time points from first release of the aortic crossclamp. *hs*, High sensitivity; *SD*, standard deviation; *RIPC*, remote ischemic preconditioning.

**Table E2:** Preoperative oxygen saturations by congenital heart defect group

Preoperative oxygen saturations	Congenital heart defect		
	TOF without RVOT stent (n = 54)	TOF with RVOT stent (n = 14)	VSD (n = 52)
<90% (cyanotic)	30 (56%)	10 (71%)	1 (2%)
≥90% (acyanotic)	24 (44%)	4 (29%)	51 (98%)
Median (IQR), %	88 [86-97]	83.5 [81-90]	98 [97-99.5]

*TOF*, Tetralogy of Fallot; *RVOT*, right ventricular outflow tract; *VSD*, ventricular septal defect; *IQR*, interquartile range.

**Table E3:** Age category by congenital heart defect group

Age	Congenital heart defect		
	TOF without RVOT stent (n = 54)	TOF with RVOT stent (n = 14)	VSD (n = 52)
<1 y	45 (83%)	8 (57%)	35 (67%)
≥1 y	9 (17%)	6 (43%)	17 (33%)
Median (IQR), mo	8.0 [5.0-10.0]	10.5 [7.0-13.0]	5.5 [3.5-14.5]

*TOF*, Tetralogy of Fallot; *RVOT*, right ventricular outflow tract; *VSD*, ventricular septal defect; *IQR*, interquartile range.

**Table E4:** Cardiac index in the first 12 hours measured using ICON in Birmingham only

Cardiac index (L/min/m^2^)	RIPC mean (SD, n)	Control mean (SD, n)	Point estimate (95% CI), *P* value∗
Baseline	3.7 (1.3, 41)	4.1 (1.3, 41)	(–0.16 to 0.59), .22
3 h	3.8 (1.4, 36)	3.7 (1.1, 38)	
6 h	3.7 (1.3, 39)	3.8 (1.4, 37)	
9 h	3.6 (1.0, 37)	3.8 (1.4, 32)	
12 h	3.6 (1.0, 35)	3.8 (1.5, 29)	

*RIPC*, Remote ischemic preconditioning; *SD*, standard deviation; *CI*, confidence interval.

∗ Average mean difference over each time point (RIPC – Control) calculated using a mixed linear regression model adjusting for baseline value. A positive difference favors the RIPC group.

## Appendix E1

### APPENDIX E1. SUPPLEMENTARY METHODS

Only patients with the most common form of TOF were included; variants such as absent pulmonary valve syndrome, pulmonary atresia with major aortopulmonary collateral arteries, or with an atrioventricular septal defect were not included. Regulatory approval from the Medicines and Healthcare products Regulatory Agency was not required because this trial was not a clinical trial of an investigational medicinal product.

### Cardiac Output Measurement

Cardiac output was measured as an exploratory end point using ICON (Osypka Medical), a CE-marked, Food and Drug Administration–approved device that uses noninvasive electrical velocimetry to provide a continuous assessment of hemodynamic parameters that has been validated in infants in the early postoperative period.E1, E2 Cardiac index was determined during the first 12 hours after reperfusion in participants undergoing surgery in Birmingham only and analyzed using mixed-effect, repeated-measures models using all available data, presented as an adjusted mean difference and 95% CI.

### Metabolic Phenotyping

Untargeted metabolomic analysis was performed applied. Tissue samples were extracted applying a biphasic extraction protocol using chloroform, methanol, and water and dried as was previously described.^E3^ Four complementary ultra high-performance liquid chromatography-mass spectrometry assays were applied to analyze each sample extract, as previously described, including the analysis of pooled QC samples and process blank samples.^E4^ Vendor format raw data files (.RAW) were converted to the mzML file format using ProteoWizard software.^E4^ Deconvolution was performed by XCMS software 4 (version 1.46.0 running in the Galaxy environment).^E4^ QC samples with a total peak area (of all features) exceeding ±25% of the median QC total peak area were removed from the data matrix. Features were retained in the data matrix if they were present in more than 90% of QC samples; had a peak area relative SD less than 30% across QC samples; and had an extract blank/mean QC area ratio of less than 5%. Features with a less than 50% detection rate over all samples were also removed. Univariate analysis was performed applying 1-way analysis of variance on PQN normalized and glog transformed data with a critical *P* value > .05 applied after correction for multiple testing applying the Benjamini-Hochberg procedure.^E5^

## Supplementary Results

The first patient was randomized on October 24, 2016, and the last participant completed follow-up on December 21, 2020.

### Coronavirus Disease 2019 Pandemic

Along with all other non–Coronavirus Disease public health studies, recruitment to the trial was suspended on March 13, 2020, due to the impact of the Coronavirus Disease 2019 pandemic on the National Health Service. During the suspension, 29 otherwise eligible patients underwent surgery at the 2 sites but had to be excluded from the trial. Recruitment recommenced on June 29, 2020, and all patients recruited subsequently were shown to be negative for severe acute respiratory syndrome coronavirus 2 with reverse-transcriptase polymerase chain reaction on 2 consecutive swabs before undergoing surgery.

### Application of the Intervention

In the RIPC arm, the intervention was applied to both thighs in 55 (92%) participants and to 1 thigh and 1 upper arm in 5 (8%) participants due to 1 lower limb being unavailable. The mean limb occlusion pressures for the 3 cycles were 152 mm Hg (±11), 156 mm Hg (±18), and 156 mm Hg (±12), respectively. Adherence to treatment allocation was achieved in 116 (96.7%) participants; no participants in the control arm received RIPC but 4 (7%) participants in the RIPC arm did not have 3 confirmed cycles of limb ischemia-reperfusion, with continual loss of arterial flow confirmed by distal pulse oximetry during each period of limb ischemia. There were no immediate or delayed limb complications due to the use of RIPC.

### Cardiac Output Measurement

Hemodynamic assessment was performed using ICON in 82 children undergoing surgery in Birmingham only. There was no difference in cardiac index in the first 12 hours between the 2 groups (Table E4). There was incomplete data recording in the postoperative period due to repeated electrode detachment or persistent movement artefact after waking, limiting its value as a routine monitoring tool in the setting of postsurgical ICU care.

### Serious Adverse Events

There were 11 serious adverse events in 9 participants: 4 in the RIPC group and 5 in the control group. These comprised 2 postcardiotomy extracorporeal life supports and 9 early surgical or catheter reinterventions, of which 3 were for residual lesions: 1 enlargement of RVOT, 1 surgical closure of residual VSD, and 1 catheter closure of residual VSD and stenting of pulmonary artery. All were assessed to be unrelated to the trial intervention and no actions were required by the sponsor.
